# Appropriate or inappropriate ICD shock; what is the post-shock rhythm?

**DOI:** 10.1007/s12471-017-1000-5

**Published:** 2017-05-17

**Authors:** B. M. van Gelder, B. ter Burg, F. A. L. E. Bracke

**Affiliations:** Department of Electrophysiology, Catharina Hospital, Eindhoven, The Netherlands

## Answer

**Fig. 1:**
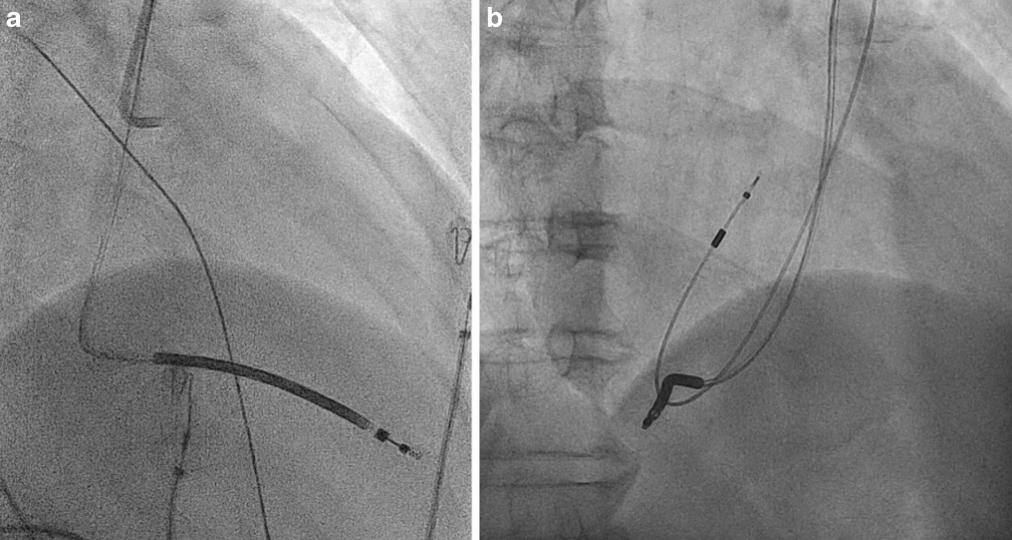
X-ray right anterior oblique view showing an apical lead position at implant (*left panel*) and displacement into the right atrium in the post-shock X‑ray (*right panel*)

At first sight, this may seem an appropriate shock for ventricular fibrillation, followed by conversion to sinus rhythm. However, the abrupt change in morphology of the RV electrogram with extreme short fibrillation sense (FS) intervals below 200 ms is not suggestive of ventricular fibrillation. The intervals are also comparable with the atrial sense (AS) and atrial sensing in the blanking period (Ab) intervals in the atrial channel. These observations suggest that the sensed arrhythmia in the ventricular channel is not ventricular fibrillation, but sensing of atrial fibrillation. This observation can only be explained by a displacement of the ventricular lead towards the right atrium. Review of the X‑rays, which at implant showed a normal RV apical position of the shock lead (Fig. 1, left panel), showed migration of the lead into the right atrium in the post-shock X‑ray (Fig. 1, right panel). It is therefore clear that the shock therapy was inappropriate.

**Fig. 2:**
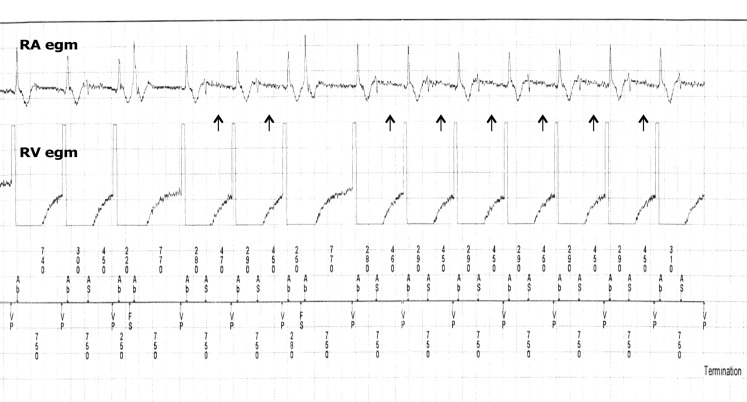
After stabilisation, the right atrial electrogram shows atrial stimulation by the ventricular lead (Vp) with subsequent antegrade conduction to the ventricle as indicated by the ventricular far-field electrogram (*arrows*) in the right atrial electrogram (As)

The post-shock right atrial electrogram showed the simultaneous termination of atrial fibrillation, which suggests ventricular pacing. However, the ventricular pace pulse is actually stimulating the atrium. This is proven by the coinciding atrial activation on the atrial lead (Fig. 2).

The effective atrial stimulation from the ventricular pace pulse is a further confirmation of the ventricular lead dislocation. The far-field R wave signal in the atrial channel indicates subsequent antegrade conduction to the ventricle (Fig. 2).

